# Regulation of gene expression by 5mC DNA methylation during the bolting process of cabbage (*Brassica oleracea* L. var. *capitata* L.)

**DOI:** 10.3389/fpls.2026.1806811

**Published:** 2026-04-30

**Authors:** Huiling Guo, Xinyu Chen, Bin Zhang, Xuehui Yao, Mu Zhuang, Limei Yang, Jialei Ji, Yong Wang, Honghao Lv, Yangyong Zhang

**Affiliations:** 1 State Key Laboratory of Vegetable Biobreeding, Institute of Vegetables and Flowers, Chinese Academy of Agricultural Sciences, Beijing, China; 2 Winter Cabbage Research and Development Center of the Yangtze River Basin, Jiayu, Hubei, China

**Keywords:** bolting, cabbage, DNA methylation, reproductive, WGBS

## Abstract

DNA methylation is an essential epigenetic modification. However, the dynamics of DNA methylation during cabbage bolting remain poorly understood. To investigate changes in DNA methylation during cabbage bolting, we performed whole-genome bisulfite sequencing (WGBS) on both vegetative leaves of non-bolting plants and reproductive leaves of bolting plants in two cabbage accessions. Across the whole genome, the DNA methylation levels on each chromosome showed a negative correlation with gene density, and the methylation levels in reproductive leaves after bolting were higher than those in vegetative leaves before bolting. We identified a total of 10,159 differentially methylated regions (DMRs). GO enrichment analysis revealed that differentially methylated genes were significantly enriched in pathways related to reproductive process and floral organ development. At the subgenome level, MF2 exhibited a higher DNA methylation advantage, and the dominance of all three subgenomes was greater in reproductive leaves than in vegetative leaves. Integrating the transcriptomic data, we found that genes with DNA methylation modifications showed markedly lower expression levels than those without methylation. Two meristem-development-related genes, *STM* and *WUS*, displayed reduced DNA methylation and significantly elevated expression during bolting, suggesting that their expression was regulated by methylation. In summary, our study revealed a comprehensive DNA methylation profile of cabbage bolting and highlighted the critical role of DNA methylation in this process. These results provide a theoretical foundation for further molecular studies on bolting in Brassicaceae plants.

## Introduction

DNA methylation is a major epigenetic modification that contributes to the epigenetic regulationof nuclear gene expression and genome stability (Robertson,2005; Slotkin and Martienssen, 2007). Recent comprehensive reviews have highlighted that epigenetic regulation-including DNA methylation, histone modifications, and non-coding RNAs-serves as a crucial mechanism for plants to respond and adapt to various environmental stresses (Abdulraheem et al., 2024). The dynamic nature of these epigenetic modifications enables plants to modulate gene expression rapidly in response to changing conditions, providing a survival advantage without altering the underlying DNA sequence. For instance, DNA methylation is involved in the response to salt stress in cotton (Yang et al., 2025), undergoes dynamic reprogramming during pathogen infection in potato (Tian et al., 2026).

The DNA methylation process is conserved in fungi, plants and mammals, and is catalyzed by DNA methyltransferases in both organisms (Bewick et al., 2019; Nai et al., 2021; Law and Jacobsen, 2010; He et al., 2011; Huang et al., 2016). In plants, DNA methylation occurs in all cytosine contexts, primarily at CG, CHG, and CHH (where H represents A, T, or C) (Zhang et al., 2006; Lister et al., 2008). The establishment and maintenance of DNA methylation are mediated by two distinct classes of methyltransferases with different biological roles. *De novo* methyltransferases are responsible for establishing methylation patterns at previously unmethylated sites, primarily through the RNA-directed DNA methylation (RdDM) pathway (Pikaard et al., 2012; Matzke and Mosher, 2014; Erdmann and Picard, 2020). In contrast, maintenance methyltransferases ensure faithful propagation of existing methylation patterns during DNA replication. These two classes also differ in their preferred cytosine contexts: *de novo* methyltransferases, such as DRM2, can establish methylation in all three sequence contexts (CG, CHG, and CHH), whereas maintenance methyltransferases act in a context-specific manner—Methyltransferase 1 (MET1) maintains CG methylation (Kankel et al., 2003; He et al., 2011), Chromomethylase 3 (CMT3) primarily catalyzes CHG methylation maintenance (Lindroth et al., 2001; Stroud et al., 2014), and CHH methylation is sustained by DRM2 or CMT2 (Huettel et al., 2006; Zemach et al., 2013; Liu et al., 2014). The demethylation process is catalyzed by demethylases, with four primary enzymes responsible for DNA demethylation in plants: REPRESSOR OF SILENCING 1 (ROS1), TRANSCRIPTIONAL ACTIVATOR DEMETER (DME), DEMETER-LIKE PROTEIN 2 (DML2), and DML3 (Gong et al., 2002; Gehring et al., 2006; Ortega-Galisteo et al., 2008).

Flowering plants achieve the transition from vegetative to reproductive growth by responding to various environmental and developmental signals. In higher plants, flowering is determined by pathways regulating flowering timing, inflorescence architecture, and floral morphogenesis, with key initiators including the florigen FLOWERING LOCUS T (FT), the antiflorigen TERMINAL FLOWER 1 (TFL1), and LEAFY (LFY). In *Arabidopsis*, FT is induced by long-day conditions and produced in the rosette leaves, then transported over long distances to the shoot apical meristem (SAM), where it converts the SAM into an inflorescence meristem (IM) to promote flowering (Zhu et al., 2016). The inflorescence meristem further differentiates to form lateral meristems (AM) and flowers. The homologous gene TFL1 in the FT family exhibits the opposite function: it inhibits flowering and maintains the vegetative developmental identity of meristems (SAM, IM, and AM) (Zhu and Wagner, 2020). Additionally, both TFL1 and FT act as co-transcription factors (co-TFs), requiring dimerization with the bZIP transcription factor FD to regulate downstream target genes (Zhu and Wagner, 2020; Zhu et al., 2020). LFY, a critical gene for inflorescence and floral development, serves as a key integrator of the developmental signals mediated by TFL1 and FT (Zhu et al., 2020).

The meristem is a multilayered structure that can be divided into central zone (CZ) and peripheral zone (PZ), with the CZ characterized by slow cell division rates and the PZ marked by rapid cell proliferation (Satina et al., 1940). The stem cells in the central zone must maintain active proliferation to sustain plant growth. CLAVATA3 (CLV3) serves as a marker for stem cell identity (Fletcher et al., 1999). The expression of *WUS* and *STM* in stem cells suppresses differentiation while activating *CLV3* expression (Brand et al., 2002; Lenhard et al., 2002). Specifically, STM binds to the *CLV3* promoter and, through WUS-STM interaction, enhances WUS binding to the same promoter. The regulation of *CLV3* expression requires the heterodimerization of WUS and STM and their simultaneous binding to two distinct sites on the *CLV3* promoter. This mechanism that ultimately maintains a constant population of stem cells (Su et al., 2020). Furthermore, STM expression depends on WUS, and WUS-activated STM expression enhances WUS-mediated stem cell activity (Lenhard et al., 2002). Simultaneously, the CLV3 protein restricts WUS expression in stem cells by binding to and modulating the activity of membrane-localized receptor complexes (Sang et al., 2022).

Cabbage (*Brassica oleracea* var. *capitata*) is a widely cultivated cruciferous vegetable crop in the *Brassica* genus, playing a vital role in achieving year-round vegetable supply. While bolting is essential for seed production in cabbage, premature bolting often leads to considerable yield loss. Therefore, understanding the molecular mechanism of bolting is crucial for regulating this process in cabbage. As a vernalization-requiring plant, cabbage bolting is induced by appropriate low-temperature exposure (Gardner and Loomis, 1952). Epigenetic modifications are considered to have a significant effect in the growth and development of eukaryotes, and the role of DNA methylation in the differentiation of the reproductive system in flowering plants has been demonstrated (Yang et al., 2022). Bolting is an important marker for the transition from vegetative growth to reproductive growth in flowering plants and a necessary process for seed maturation. However, it is not clear whether 5mC methylation is also involved in the regulation of cabbage bolting.

In this study, we conducted integrated DNA methylome and transcriptome analyses on two cabbage genotypes under different vernalization conditions. Our results demonstrate that DNA methylation is closely associated with gene expression during bolting, and the differentially methylated genes (DMGs) between vegetative leaves to reproductive leaves were significantly enriched in pathways related to floral organ development and hormone signaling. Integrated analysis of DNA methylome and transcriptome data revealed altered DNA methylation patterns and significant differential expression of WUS and STM, two key regulators of meristem identity. These findings suggest that DNA methylation may participate in regulating meristem development during cabbage bolting, thereby influencing bolting time. Our study investigate the divergent DNA methylation regulation underlying early and late bolting in cabbage, and identifies two critical methylated genes that govern leaf-bolting meristem development. These findings provide epigenetic insights into the molecular mechanisms of cabbage bolting.

## Materials and methods

### Plant materials and growth conditions

On September 1, 2022, two different genotypes of cabbage bolt-resistant materials JY81 and bolt-sensitive materials Milan were sown and grown in a greenhouse (12 hours light at 25 °C/12 hours dark at 20 °C). After four weeks, the seedlings were transplanted into pots. On December 2, the plants were moved to a vernalization greenhouse and cold frames, respectively, for vernalization. The materials vernalized in cold frames bolted normally, and the young bolting leaves of JY81 and Milan were collected as RL1 and RL2, respectively; the materials vernalized in the greenhouse did not bolt normally, and the inner leaves of JY81 and Milan were collected as VL1 and VL2.

### RNA−seq library construction and transcriptome analysis

Early bolting leaves from both bolt-resistant materials JY81 and bolt-sensitive materials Milan were sampled, followed by total RNA extraction using Trizol (Invitrogen) reagent. RNA concentration and purity were subsequently assessed by spectrophotometry. Two biological replicates of RNA-seq libraries were constructed for each group. Sequencing was performed via an BGI platform (DNBSEQ-T7) at the genedenovo Company (Guangzhou, China), and paired reads with an average length of 150 bp were generated. The raw high-throughput sequencing data (FASTQ file) were initially assessed for quality using FastQC. Adapter sequences and poor-quality reads were removed using Trimmomatic v0.38. After data quality control, the clean reads were mapped to the B. oleracea genome (v2.0) via HISAT2 (v2.0.4) (Kim et al., 2019) with parameters: hisat2 -p 8 --dta -x. Removal of non-nuclear comparisons from SAM files using SAMTools v1.14, and the resulting BAM files were sorted and PCR duplicates removed with the parameter “-b -q 10”. The expression abundance of mapped reads was quantified using StringTie (v2.2.1) with the parameter: stringtie input-file -G gff -p 8 -e. EdgeR package (v3.40.2) was used for differential gene detection, with a |Log_2_fold change| > = 1.00 and adjusted pvalue < = 0.01. The gene counts were converted to FPKM using GenomicFeatures (1.50.4). The GO database “THE GENE ONTOLOGY RESOURCE (https://geneontology.org/)” was utilized.

### Bisulfite sequencing library construction and whole−genome bisulfite sequencing

The samples used for BS-seq (bisulfite sequencing) library construction were the same as those for RNA-seq library preparation. The leaf tissues were used for genomic DNA extraction via a CTAB-based protocol with two biological replicates. Quantification was performed with an Agilent 2100 spectrophotometer. Bisulfite treatment (EZ DNA Methylation-Gold Kit, Zymo, D05005), library construction (TruSeq DNA Methylation Kit, Illumina, EGMK91396), and sequencing (iIIumina Novaseq6,000) were performed at Oebiotech Company (Shanghai, China). Adapter sequences and poor-quality reads were removed using Trimmomatic v0.38. Bismark (v2.5.1) (Krueger and Andrews, 2011) was used to map the clean data to the *B. oleracea* genome (v2.0), the parameters were Bismark --genome fasta_direc -1 -2, then remove the repeat reads with deduplicate_bismark. The DNA methylation sites were extracted by bismark_methylation_extractor with the parameters: bismark_methylation_extractor -p --gzip --bedGraph --buffer_size 10G --cytosine_report --comprehensive --genome_folder fasta_direc. Reads with coverage >10 were selected as confidently detected cytosine sites (C-sites), followed by differential methylation analysis using the R package DSS. The parameters used for DSS were a smoothing window size of 500 bp, a minimum read coverage threshold of 10× per cytosine site, and DMRs were defined as regions containing at least 3 CpG sites with a minimum length of 50 bp, an absolute methylation difference ≥ 0.1, and a P < 1e-5.

## Result

### Genome-wide DNA hypermethylation occurs during cabbage bolting

To detect the genome-wide DNA methylation variation that occurred during cabbage bolting, we simultaneously constructed bisulfite sequencing (BS-Seq) libraries form both vegetative leaves (VL) and reproductive leaves (RL) of bolt-resistant genotype ‘JY81’ and bolt-sensitive genotype ‘Milan’. Each material was sequenced with two biological replicates. For each sample, 4.64M-5.27M paired-end clean reads (read length = 150M) were produced, at least 82.88% of reads are mapped the JZS2.0 genome (**Supplementary Table 1**). We performed correlation analysis and principal component analysis (PCA) for each sample, which showed that the correlation was higher within the two breed groups and that the two replicates of the same tissue tended to cluster together (**Supplementary Figures 1**, **2**).

**Figure 1:**
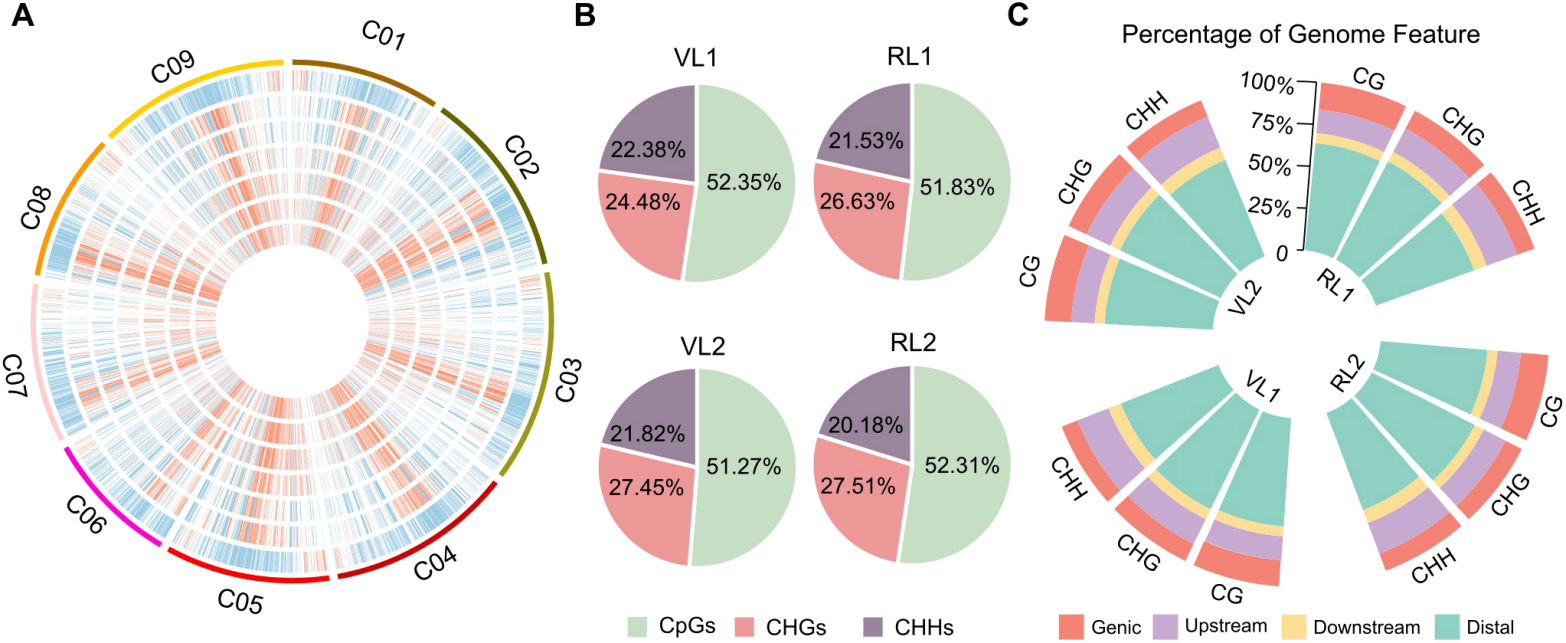
The distribution patterns of DNA cytosine methylation levels in the cabbage genome. **(A)** Circos plot shows the landscape of DNA methylation across the nine chromosomes of cabbage during vernalization. From the outermost to the innermost ring: chromatin gene density distribution, methylation levels of VL1-CpG, RL1-CpG, VL1-CHG, RL1-CHG, VL1-CHH and RL1-CHH. **(B)** The average rates of methylated cytosines (mC) in the three contexts in the cabbage genome for late bolting leaf and early bolting leaf. **(C)** Distribution of mCs on different genomic features.

We depicted the DNA methylation landscape across the nine chromosomes of cabbage during bolting. The modification levels of all three contexts CG, CHG, and CHH were highly modified in regions with low gene density and lower levels in regions with high gene density (**Figure 1A**; **Supplementary Figure 3**). Next, we calculated the percentage of DNA methylation contexts and found that the CpG context (~ 52%) was the most frequently methylated in the cabbage genome (**Figure 1B**). In the whole genome, the distribution of CG, CHG, and CHH methylation was similar between VL and RL. DNA methylation was highest in distal regions, followed by gene body regions, while methylation levels were lowest in the upstream (TSS -2000 bp) and downstream (TTS + 1000 bp) regions (**Figure 1C**). These results suggest that the pattern of methylation distribution in cabbage is similar to that observed in other species (Yang et al., 2022; Kong et al., 2025).

### DNA methylation plays a crucial role in the bolting process of cabbage

**Figure 2:**
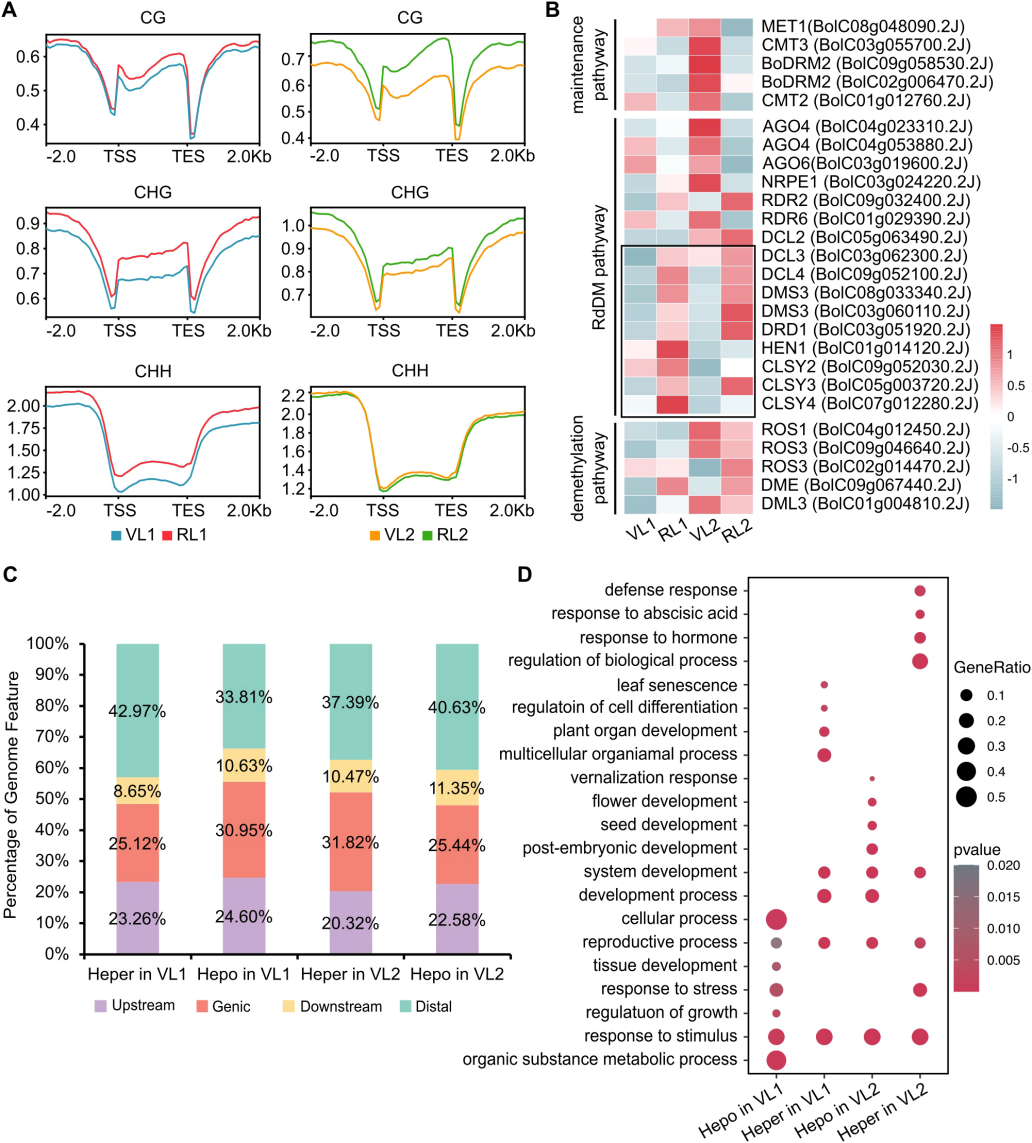
Differential methylation analysis of bolting and non-bolting. **(A)** Distribution of DNA methylation levels across the upstream 2 Kb, gene body, and downstream 2 Kb regions of genes. “TSS” indicates the transcriptional start site, and “TES” indicates the transcription end sites. **(B)** Heatmap showing transcriptome levels of key component genes of the maintenance pathways, RdDM pathway and demethylation pathway in VL1, RL1, VL2, RL2. The rectangular box contains methylases showing expression changes consistent with methylation level changes. Scale bar represents the z-score of the normalized expression values. **(C)** The distribution characteristics of differentially modified regions (DMRs) in the cabbage genome. **(D)** GO enrichment analysis of differentially methylated genes (DMGs).

We calculated average methylation levels for every 100 bp interval of each gene and, encompassing 2 Kb upstream and downstream flank regions, this analysis revealed that CG and CHG methylation levels increased greatly in the 5′ and 3′ regions and slightly in gene bodies. We observed consistent patterns of methylation changes in both ‘JY81’ and ‘Milan’. The levels of mCG, mCHG, and mCHH in cabbage were increased during the reproductive stage compared to the vegetative stage, except for CHH methylation in RL2 (**Figure 2A**). To investigate the mechanism of DNA hypermethylation during bolting process, we examined all the expression of DNA demethylation and DNA methylation. In Arabidopsis, the RdDM pathway plays a crucial role in genome-wide DNA methylation reprogramming (Zhang et al., 2018). We found that genes in the RdDM pathway, such as *DCL3*, *DCL4*, *DMS3*, *DRD1*, *HEN1*, *CLSY2*, *CLSY3* and *CLSY4* showed higher expression in RL1 and RL2 compared to VL1 and VL2, suggesting enhanced RdDM activity during bolting process in *B. oleracea* (**Figure 2B**).

To investigate the effect of modification differences on bolting time, differential DNA methylation analysis was performed. In RL1, we identified 19,984 hypermethylated differentially methylated locus (hyper-DMLs) and 3,792 hypomethylated DMLs (hypo-DMLs), which were mapped to 4,609 hypermethylated differentially methylated regions (hyper-DMRs) and 607 hypomethylated differentially methylated regions (hypo-DMRs), respectively. In RL2, 19,547 hyper-DMLs and 14,235 hypo-DMLs were identified, corresponding to 2,858 hyper-DMRs and 2,361 hypo-DMRs, respectively.

We investigated the genomic composition of the DMRs on the whole genome. Approximately 20.31%- 24.6% were located upstream (<2Kb from TSS) of genes, 25.12%- 31.84% within gene body, 8.65%- 11.35% downstream (<1Kb form TTS) of genes, and 33.81%- 42.97% in distal region (>2Kb from TSS, >1Kb form TTS). Among these, gene body and intergenic region contained the highest proportions of DMRs, while upstream and downstream regions showed relatively lower distributions (**Figure 2C**). Annotation of these differentially methylated regions yielded 4,020 hyper differentially methylated genes (hyper-DMGs) in RL1 and 1,353 hyper-DMGs in VL1, 4,757 hyper differentially methylated genes (hyper-DMGs) in RL2 and 3,655 hyper-DMGs in VL2. GO enrichment analysis of these genes found that mainly play a role in the process of development and resistance to adversity during growth, these pathways mainly include reproductive process, organ development, leaf morphology, vernalization response and flower development (**Figure 2D**). Genes associated with reproductive processes include *WUS*, *HEC2*, *FLC*, *STM*, *AGO9*, among others. These genes have been demonstrated in plants such as *Arabidopsis* to participate in regulating cell fate, influence the development of reproductive meristems, and control flowering time (Shindo et al., 2006; Scofield et al., 2007; Schuster et al., 2015; Zhao et al., 2017; Bradamante et al., 2024).

### MF2 holds the dominant advantage in DNA methylation across the three subgenome

**Figure 3:**
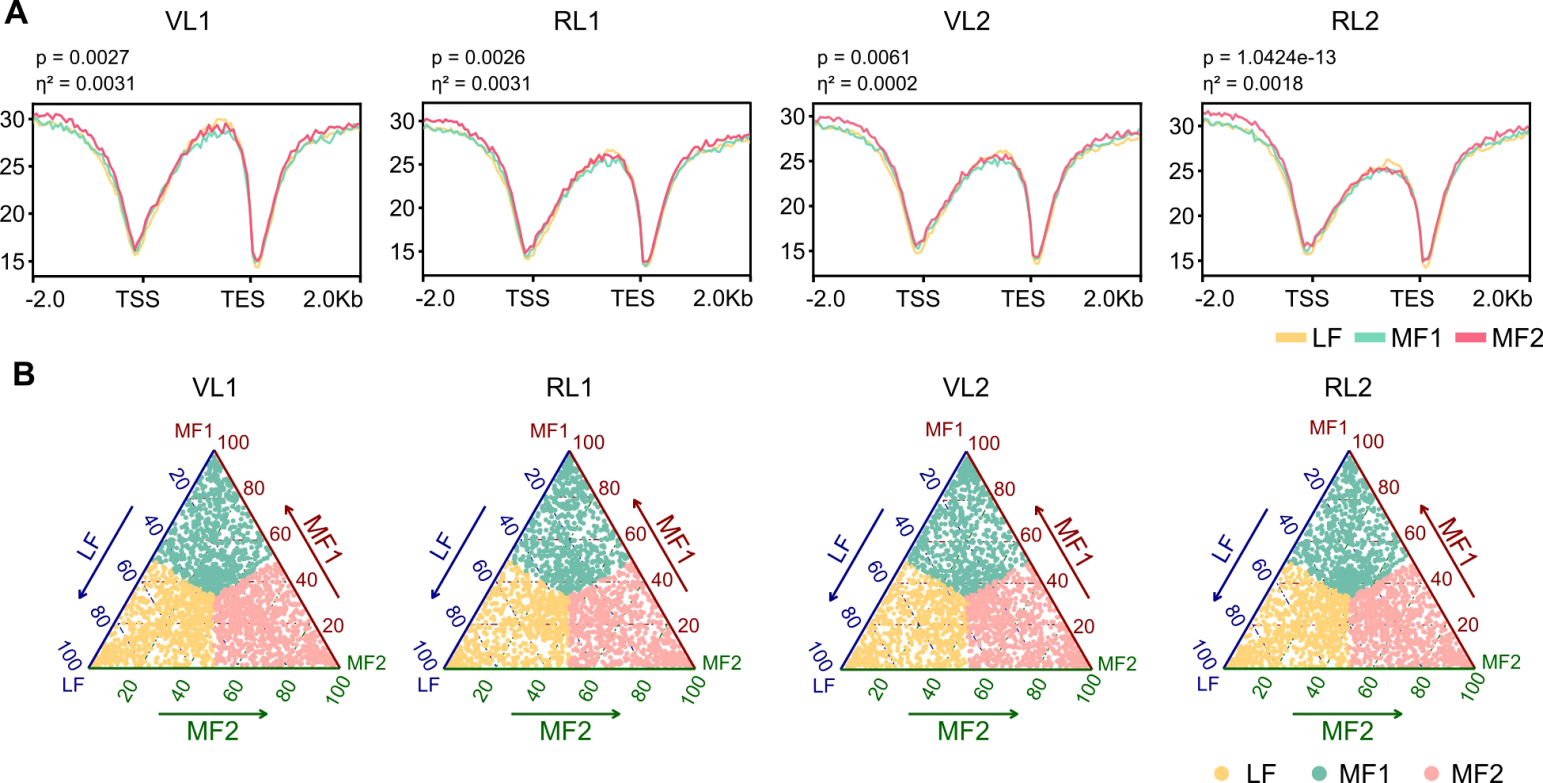
MF2 holds the marginal dominant advantage in DNA methylation. **(A)** Methylation profiles in subgenome genes. The mean level for each 50-bp interval is plotted. Significance for each pairwise comparison was calculated by Kruskal-Wallis test. In the upper left corner of the figure are the p-value and effect size. **(B)** Ternary plot showing DNA methylation bias of the syntenic genes from three subgenomes.

Due to a unique whole genome triplication (WGT) event in Brassica species, which resulted in three subgenomes (LF, MF1, MF2), duplicated genes may exhibit functional redundancy leading to gene loss or evolve new functions and be retained (Cheng et al., 2012). This process may cause functional bias among the three subgenomes. To investigate whether DNA methylation exhibits difference at subgenomes, we analyzed DNA methylation modifications in the subgenomes of different stage. Our finding indicated that the DNA methylation levels in MF2 across the whole genome of all samples were higher than that in LF and MF1 (p < 0.01, effect size < 0.01), but there was no significant difference between LF and MF1 (**Figure 3A**).

To further investigate the subgenomic dominances of DNA methylation, we conducted the DNA methylation bias analysis by ternary plotting. LF, MF1, and MF2 showed 647, 621, and 717 biased methylated genes in the reproductive leaves of JY81, respectively, while the corresponding numbers in vegetative leaves were 530, 534, and 589. Similarly, in Milan, LF, MF1, and MF2 exhibited 776, 734, and 866 biased methylated genes in reproductive leaves, respectively, while the corresponding numbers in vegetative leaves were 760, 735, and 844 (**Figure 3B**). Our analysis revealed that across all samples, MF2 exhibited more biased methylated genes than LF and MF1, indicating that DNA methylation holds a dominant advantage in MF2. Furthermore, in both the JY81 and Milan varieties, the number of methylated genes in reproductive leaves was consistently higher than in vegetative leaves across the three subgenomes (**Figure 3B**), suggesting that the bias of DNA methylation increases during the bolting process.

### DNA methylation status is associated with gene expression levels

To explore the transcription alterations throughout the bolting difference of cabbage, we conducted an RNA-seq assay. After data from each sample were filtered and subjected to quality control procedures, reliable and high-quality sequence data among the two biological replicates were obtained.

**Figure 4:**
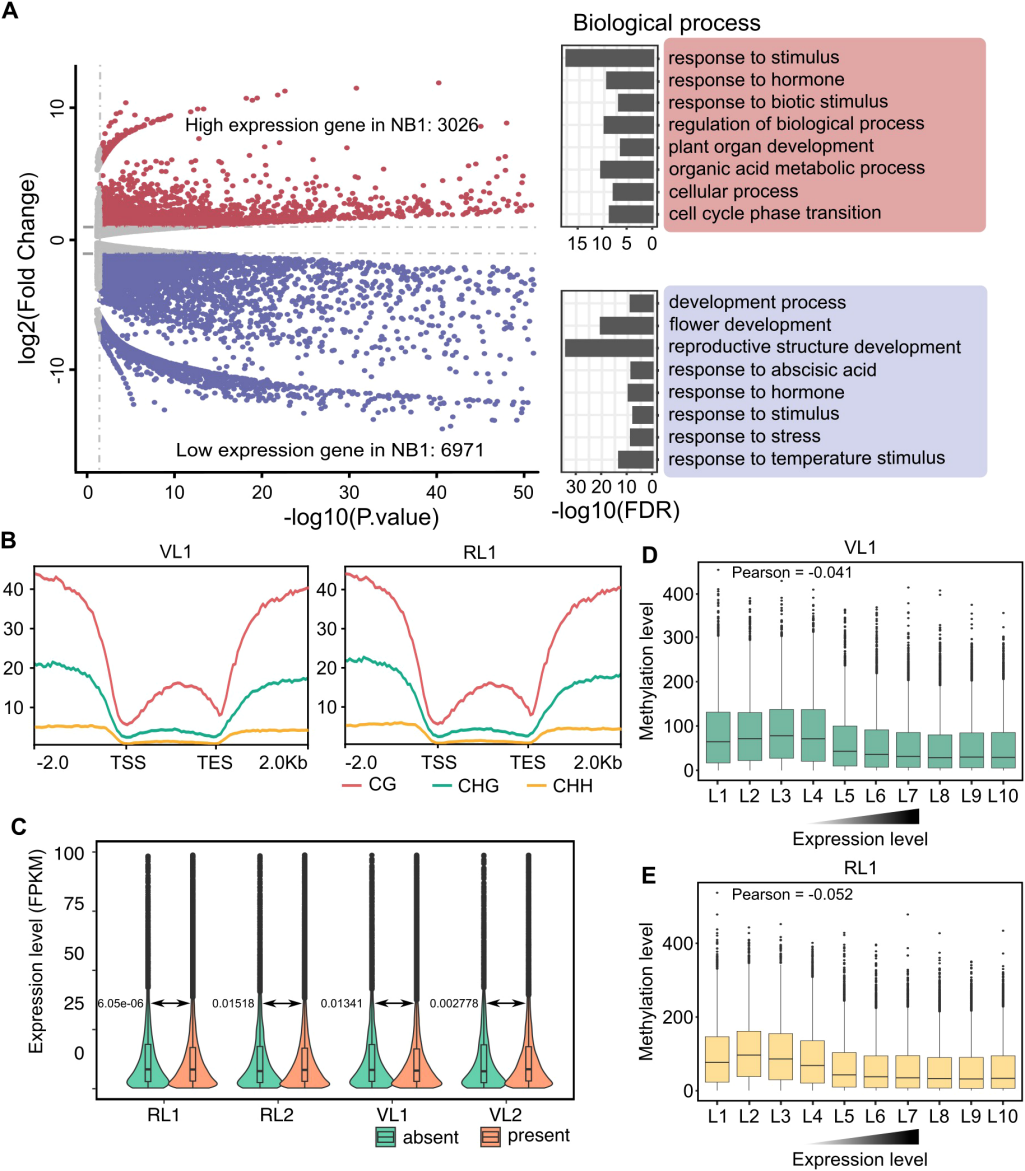
Relationship between DNA methylation and gene expression in cabbage during vernalization. **(A)** Analysis of differentially expressed genes of BL1 and VL1 and GO enrichment analysis. **(B)** Distributions of CG, CHG, CHH methylation levels in differential expression genes. **(C)** Violin plot shows the expression of genes with 5mC (present) and without 5mC (absent) across the four samples. **(D-E)** Box plot shows the relationship of different expression level (FPKM) and methylation level. Calculate the Pearson correlation coefficient between methylation levels in the promoter region and gene expression levels.

Compared to VL1, there were 3,026 up-regulated genes and 6,971 down-regulated genes in RL1. The up-regulated genes showed enrichment in response to stimulus, response to hormone, response to biological process, organic acid metabolic process and plant organ development. Meanwhile, down-regulated genes were overrepresented in reproductive structure development, flower development, response to temperature stimulus and response to abscisic acid. Correspondingly, between VL2 and RL2, there were 2621 up-regulated genes and 7410 down-regulated genes in RL1. The up-regulated genes enriched in plant organ development, cell division, response to stress, tissue development and response to stress. Conversely, the down-regulated genes were enriched in response to abscisic acid, reproductive process, floral organ development, response to cold and response to hormone (**Figure 4A**; **Supplementary Figure 4**). These results reflect that the different expression genes between bolting and non-bolting have high correlation with bolting and flowering. We further analyzed the methylation levels of differentially expressed genes, which showed highest levels of CG methylation, medium levels of CHH methylation and lowest levels of CHG methylation (**Figure 4B**; **Supplementary Figure 5**). This result may suggest that the effect of CG methylation on gene expression in cabbage bolting was the greatest.

To investigate the role of DNA methylation in gene expression associated with bolting difference, we analyzed the relationship between the degree of methylation and gene expression levels, we examined whether expressed genes across the genome exhibited DNA methylation modifications. Approximately 79.4-88.2% of the genes showed DNA methylation modifications, and the expression levels of genes modified by 5mC were generally lower than those of unmodified genes (**Figure 4C**).

After sorting genes by expression level and dividing them into ten equal groups, we found that methylation levels decreased as expression levels increased. Furthermore, the Pearson correlation coefficient between gene expression levels and methylation levels confirmed a negative correlation (**Figures 4D, E**; **Supplementary Figure 6**). These results indicate that DNA methylation affects the process of gene expression.

### Changes in DNA methylation levels influence the expression of vernalization pathway-related genes

**Figure 5:**
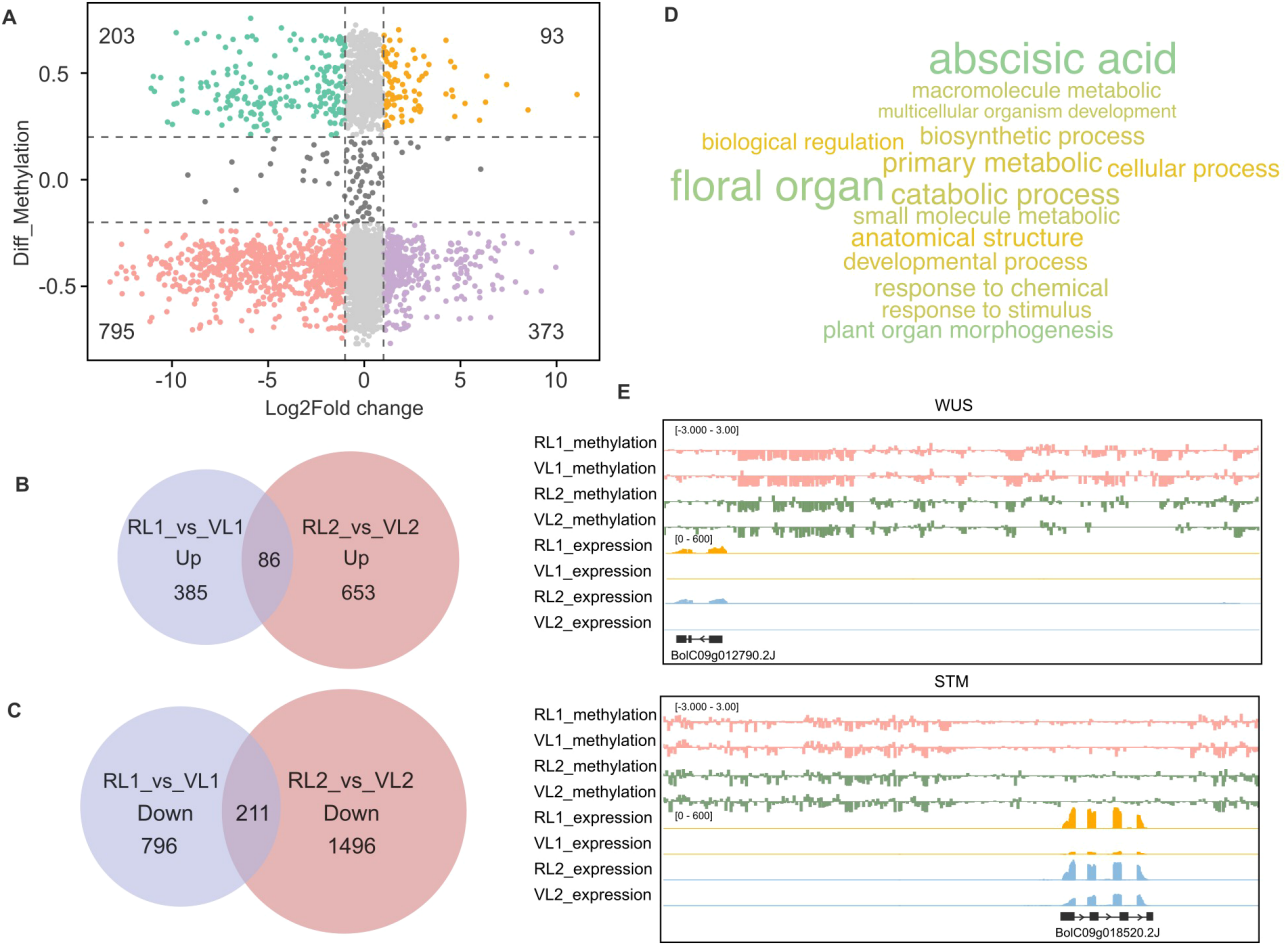
DMR-mediated DEGs related to the vernalization process of cabbage. **(A)** Number of DMR-mediated DEGs of RL1 vs VL1 (Log2Fold change ≥ |1| & Diff methylation level ≥ |0.2|). **(B, C)** Venn plot shows the overlap genes of the DMR-mediated up-regulated DEGs and DMR-mediated down-regulated DEGs in RL1_vs_VL1 and RL2_vs_VL2. **(D)** Word cloud map shows the functional enrichment pathway of the overlap gene in Figure **(E)** IGV visualization of methylation level and expression level of the *WUS* and *STM* genes.

To analyze how DNA methylation affects the bolting time of cabbage, and further identify genes that exhibit both expression differences and DNA methylation differences between bolting and non-bolting materials. The results showed that compared to the bolting material RL1, the non-bolting material VL1 had 1478 differentially expressed genes (DEGs) associated with differential methylation, including 98 hyper-up, 209 hyper-down, 373 hypo-up, and 798 hypo-down genes. Similarly, compared to the bolting material RL2, the non-bolting material VL2 had 2446 DEGs linked to differential methylation, consisting of 333 hyper-up, 715 hyper-down, 406 hypo-up, and 992 hypo-down genes (**Figure 5A**; **Supplementary Figure 7**). By taking the intersection of upregulated and downregulated genes with altered DNA methylation in the two materials, 86 common upregulated genes and 211 common downregulated genes were identified (**Figures 5B, C**). These upregulated genes are associated with various biological pathways, with the floral organ development pathway being the most prominent (**Figure 5D**). Analysis of genes related to this pathway revealed that the DNA methylation levels of *WUS* and *STM*, which regulate meristem and shoot apex development (Lenhard et al., 2002), decreased during the bolting process, while their gene expression increased significantly (**Figure 5E**). This suggests that such changes may contribute to bolting after vernalization.

## Discussion

Bolting is an epigenetically regulated mechanism through which plants have evolved to optimize their timing for reproduction, a process critical for crop production (Jean Finnegan et al., 2010). However, for *Brassica oleracea*, the global dynamic regulation of DNA methylation in response to bolting has yet to be sufficiently clarified. In this study, we performed WGBS to generate a map of DNA methylation at a single base resolution for cabbage subjected to premature bolting. Analysis of global DNA methylation levels revealed that reproductive leaves exhibited significantly higher methylation than vegetative leaves, which aligns with findings from studies on DNA methylation during vernalization in orchardgrass (Yang et al., 2022). Differential DNA methylation analysis identified 19,984 hyper-DMLs and 3,792 hepo-DMLs in the reproductive leaves of JY81, while 19,547 hyper-DMLs and 14,235 hypo-DMLs were identified in the reproductive leaves of Milan. In both materials, the number of hyper-DMLs in reproductive leaves exceeded that of hypo-DMLs, which aligns with the genome-wide distribution pattern of methylation sites between reproductive and vegetative leaves. However, compared to Milan, the number of hypermethylated sites in JY81 was significantly greater than that of hypomethylated sites, which may be attributed to the higher bolting resistance of JY81 compared to Milan.

Brassica species have undergone an additional whole-genome triplication (WGT) event following their divergence from *Arabidopsis thaliana*. As a result, the Brassica genome comprises three subgenomes, each harboring multiple homologous gene copies (Cheng et al., 2016). This structure adds a layer of complexity to epigenetic and gene expression regulation, thereby influencing the molecular mechanisms of bolting in cabbage. Our findings indicate that the coverage of DNA methylation reads in the MF2 subgenome is slightly higher than in both LF and MF1 across all four samples. And MF2 exhibited a higher number of biased methylated genes than LF and MF1 across all samples. The differences in methylation levels among subgenomes in cabbage were relatively small, consistent with previous studies which showed no obvious negative relationship between methylation level and subgenome dominance (Zhang et al., 2023). This suggests that methylation alone is insufficient to establish subgenome expression dominance, which instead arises from the combined action of methylation and other epigenetic modifications. DNA methylation not only maintains genome stability but also contributes to the regulation gene expression (Chan et al., 2005). In rice and apples, methylation in the promoter region suppresses gene expression, while methylation in the gene body promotes gene expression (Li et al., 2012; Xu et al., 2018). However, studies in *Arabidopsis* have shown that DNA methylation has a limited impact on gene expression (Meng et al., 2016). In our research, CG DNA methylation plays a major regulatory role in gene expression during bolting, while CHG and CHH have relatively minor effects on gene expression during this process. Among the expressed genes, 79.4- 88.2% exhibited DNA methylation modifications, and the expression levels of methylated genes were lower than those of unmethylated genes. This indicates that, at the whole-genome level, DNA methylation exerts a negative regulatory effect on gene expression. DNA methylation does not act in isolation; it often interacts with histone modifications to regulate chromatin state and gene expression. Studies in mammalian systems have revealed that histone methyltransferases such as SETD2 can be upregulated in response to cellular stress and DNA damage (Jemaà, 2024). In plants, the cross-talk between DNA methylation and histone H3K9me2 or H3K27me3 is well-documented. The hypermethylation observed during cabbage bolting may be accompanied by changes in histone methylation patterns, particularly at loci such as FLC, which is known to be regulated by both H3K27me3 and H3K36me3 during vernalization (Milec et al., 2014). Application of the DNA methyltransferase inhibitor 5-Azacytidine can promote flowering in vernalization-sensitive *Arabidopsis* and winter wheat (Burn et al., 1993; Kondo et al., 2007).

Reproductive is the result of plants perceiving low temperature and undergoing vernalization, in which epigenetic regulation plays a key role (Comai, 2023). Cold tolerance and vernalization response are intimately connected in many plant species, as both involve sensing and responding to low temperatures. QTL mapping studies in wild rice have identified candidate genes for cold tolerance at the germination stage (Pan et al., 2023), revealing genetic loci that may also influence developmental responses to cold. In cabbage, the vernalization requirement for bolting suggests that cold sensing mechanisms are coupled with epigenetic regulation. In *Arabidopsis*, DNA methylation levels increase from the meristem stage to early flowering, followed by a decrease from early to late flowering stages (Yang et al., 2015). In cabbage, the DNA methylation, levels decrease during the reproductive reflect a broader cold-responsive epigenetic program that affects both stress tolerance and developmental timing.

After vernalization, plants initiate bolting through the formation of new stems from meristems. Our analysis revealed that the expression levels of both *WUS* and *STM* were significantly elevated after vernalization, while the DNA methylation levels of these two genes were higher after vernalization. The regulation of STM and WUS expression likely involves complex transcription factor networks that integrate developmental and environmental signals. In Panax ginseng, MYB transcription factors have been shown to respond to jasmonate signaling and regulate key biosynthetic genes (Liu et al., 2019). Similarly, MYB and bHLH transcription factors may mediate the expression of meristem regulatory genes in cabbage, with DNA methylation potentially modulating transcription factor accessibility to target loci. The reduced methylation at STM and WUS promoters during bolting may facilitate binding of activating transcription factors, a hypothesis that warrants experimental testing.

The establishment and maintenance of DNA methylation patterns are orchestrated by specific methyltransferases whose expression may themselves be regulated during developmental transitions. Drawing parallels from insect systems where microRNAs modulate methyltransferase activity to control metamorphosis (Song et al., 2024), it is plausible that cabbage bolting involves regulatory networks that coordinate methyltransferase expression with the methylation changes observed at loci such as STM and WUS. Future work should examine whether expression of MET1, CMT3, and DRM2 correlates with the genome-wide hypermethylation observed during bolting.

Stem cell regulation in plants must integrate environmental signals, including stress, with developmental programs. Studies in animal systems have demonstrated that toxins such as deoxynivalenol can influence stem cell regeneration through the Hippo signaling pathway (Liao, 2025). While plants lack a canonical Hippo pathway, they possess analogous signaling modules that coordinate stress responses with meristem activity. The DNA methylation changes observed at STM and WUS during bolting may represent a point of integration between vernalization signals and stress-responsive pathways, ensuring that the transition to reproductive growth occurs under favorable conditions.

Beyond canonical DNA methyltransferases, methyltransferase-like proteins have emerged as important regulators of epigenetic processes in diverse organisms. In mammalian systems, methyltransferase-like 16 has been shown to drive disease progression through epigenetic suppression of target genes (Peng et al., 2025). The Brassica genome contains numerous methyltransferase-like genes whose functions remain unexplored. Some of these may contribute to the methylation dynamics observed during cabbage bolting, either through direct effects on DNA methylation or through modification of non-histone proteins that influence chromatin state.

In conclusion, our study provides a comprehensive genome-wide DNA methylation landscape associated with bolting in cabbage, revealing key epigenetic regulatory features such as subgenome methylation dominance and methylation-mediated repression of meristem development genes including STM and WUS. Understanding the molecular mechanisms governing bolting is not only of fundamental biological interest but also holds significant agronomic and economic importance. Bolting the transition from vegetative to reproductive growth is a critical developmental event in Brassicaceae crops, directly influencing yield, quality, and harvest timing. Premature bolting, for instance, can severely compromise the market value of cabbage and related vegetables, leading to substantial economic losses. Therefore, the epigenetic regulatory mechanisms uncovered in this study offer potential targets for molecular breeding strategies. By manipulating DNA methylation at key regulatory loci such as STM and WUS, it may be possible to fine-tune bolting timing to better suit agricultural production needs. More broadly, our findings contribute to a deeper understanding of reproductive development in plants, providing a valuable epigenetic framework that can be extended to other Brassicaceae crops of agricultural significance.

## Data Availability

The datasets presented in this study can be found in online repositories. The names of the repository/repositories and accession number(s) can be found below: CNCB with the accession number PRJCA057865 (https://ngdc.cncb.ac.cn/).
