# Supplementary material for: Regulation of gene expression by 5mC DNA methylation during the bolting process of cabbage (Brassica oleracea L. var. capitata L.)
Source: Front Plant Sci. 2026 Apr 30;17:1806811. doi: 10.3389/fpls.2026.1806811 (PMC13171550; doi:10.3389/fpls.2026.1806811)
Supplement: Supplementary file 1 [file DataSheet1.docx]

**
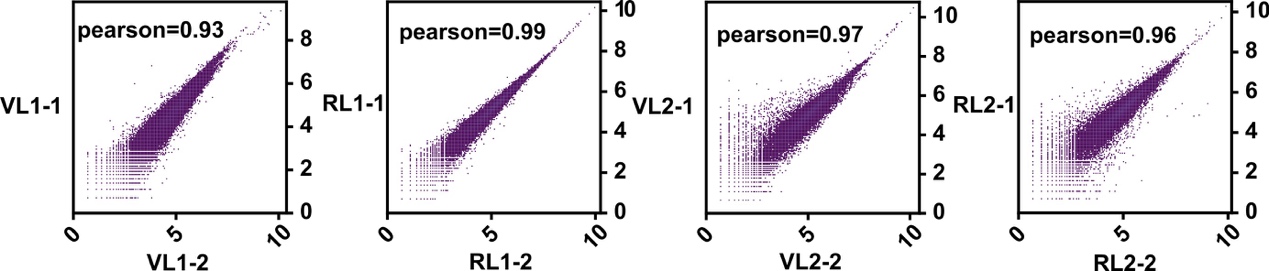
Figure S1**. Scatter plot shows the Pearson correlation coefficient between biological replicate samples at the RL1, VL1, RL2 and VL2.


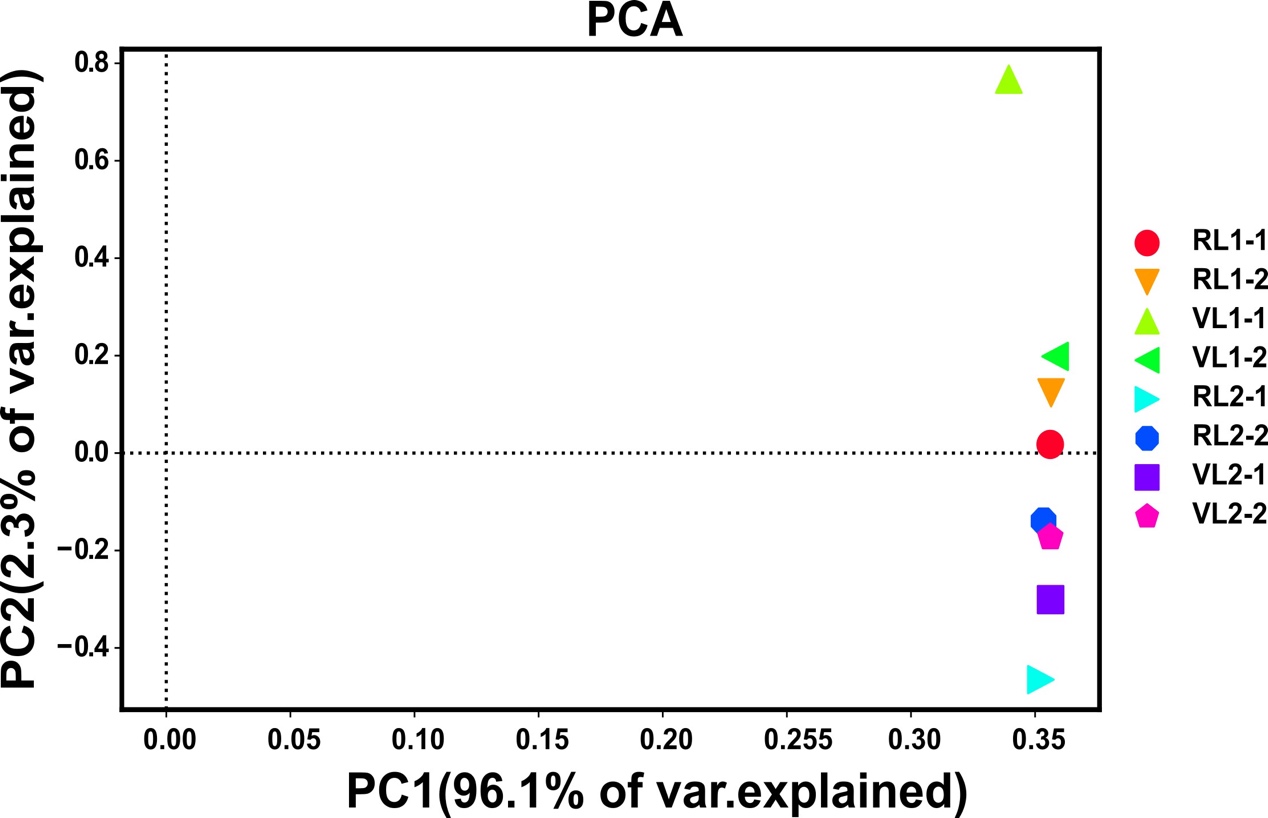


**Figure S2**. Principal component analysis for BS-seq.


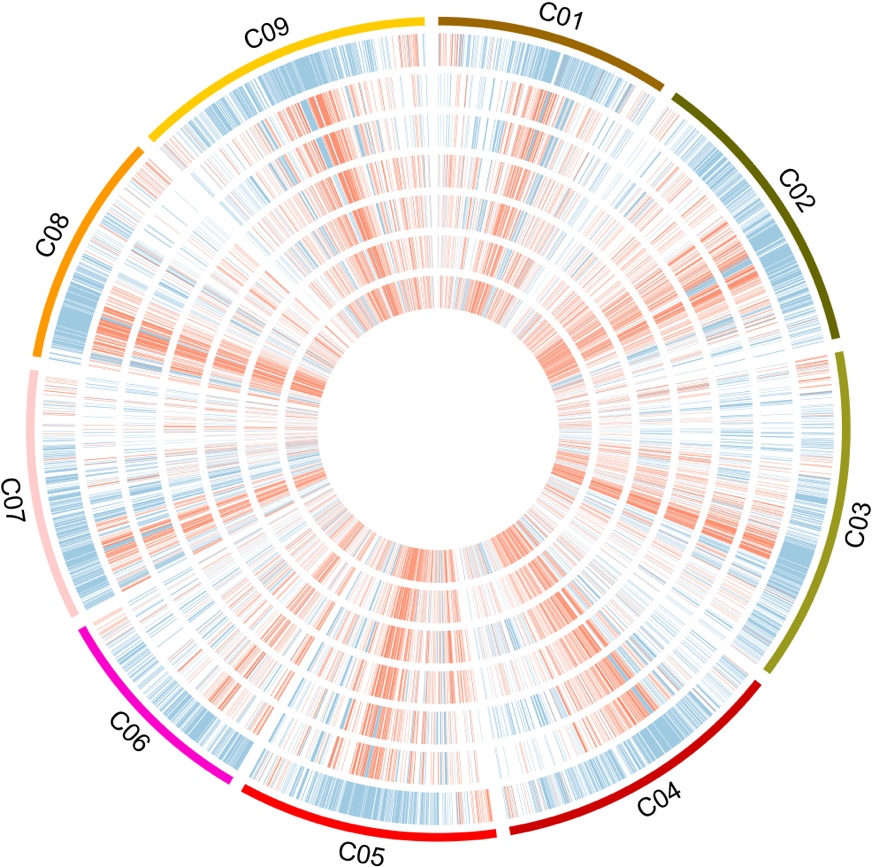
**Figure S3**. Circos plot shows the landscape of DNA methylation across the nine chromosomes of cabbage during vernalization. From the outermost to the innermost ring: chromatin gene density distribution， methylation levels of RL2-CpG, VL2-CpG, RL2-CHG, VL2-CHG, RL2-CHH and VL2-CHH.


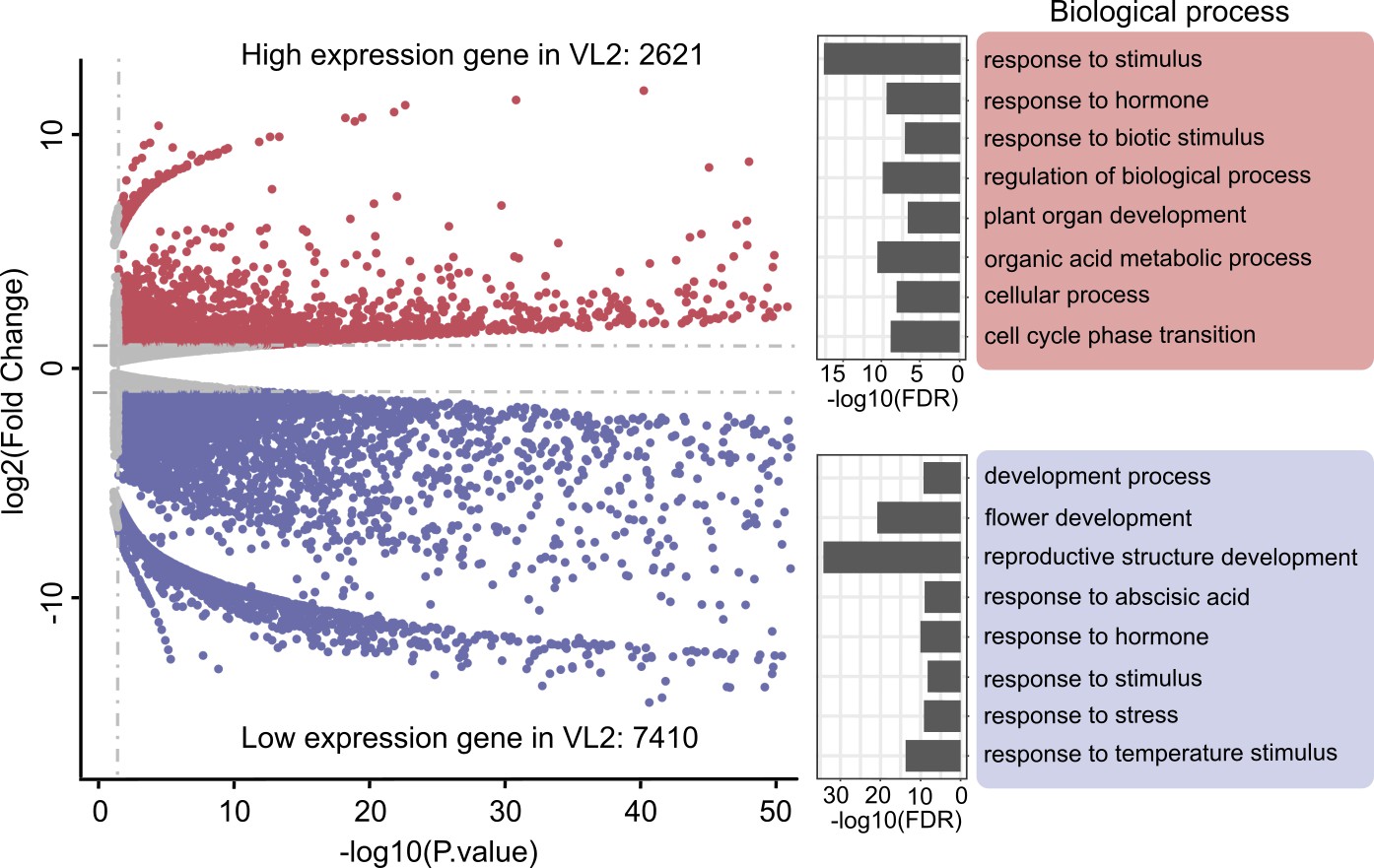


**Figure S4**. Analysis of differentially expressed genes of RL2 and VL2 and GO enrichment analysis.

**
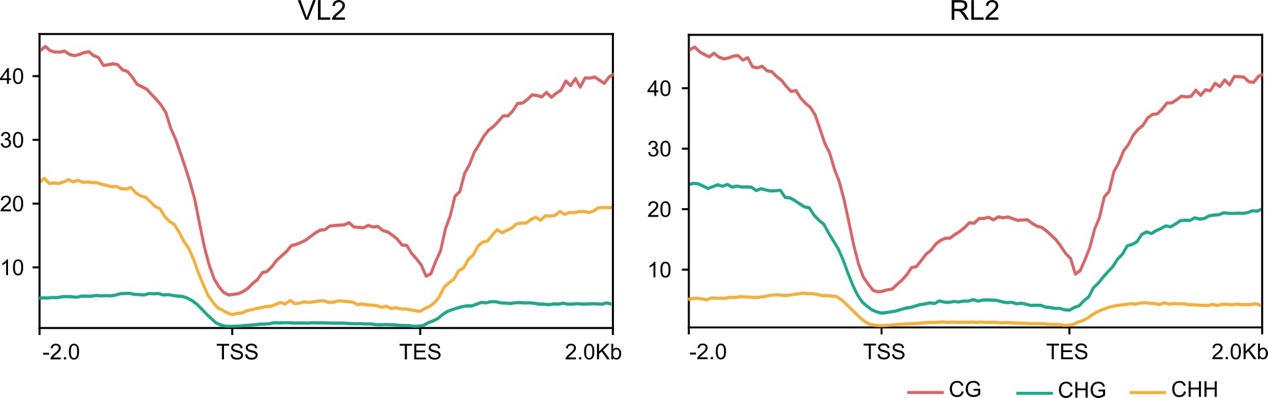
Figure S5**. Distributions of CG, CHG, CHH methylation levels in differential expression genes.


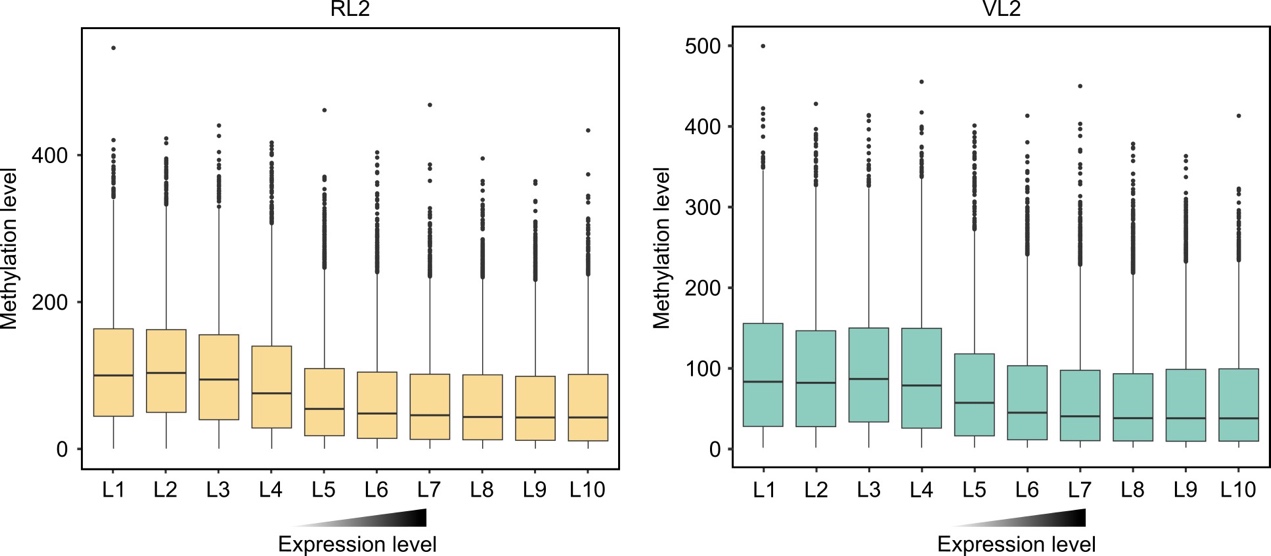


**Figure S6**. Box plot shows the relationship of different methylation level and expression level (FPKM).


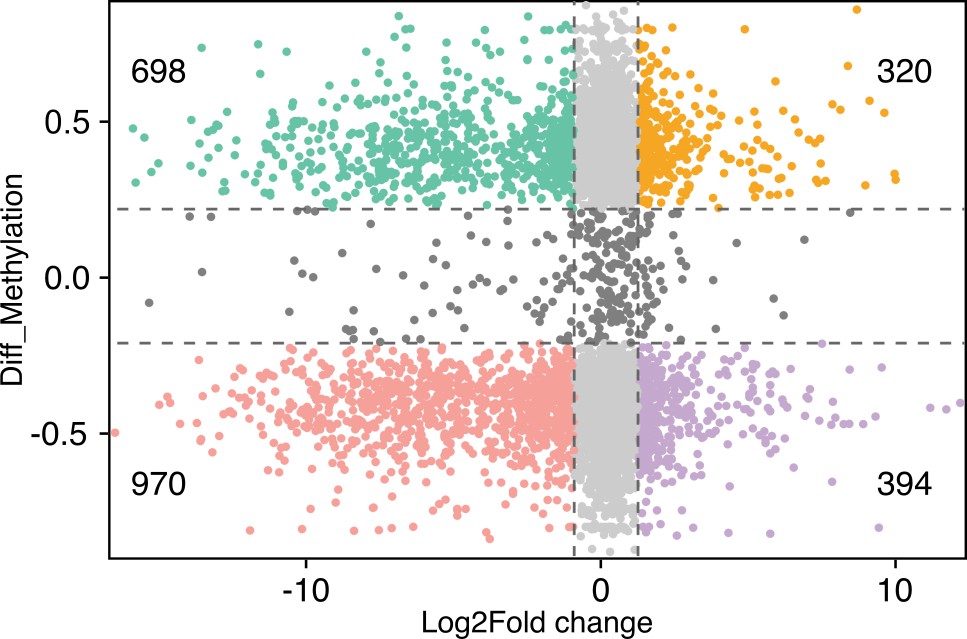


**Figure S7**. Number of DMR-mediated DEGs of RL2 vs VL2 (Log2Fold change ≥ |1| & Diff methylation level ≥ |0.2|).
